# Five-year outcomes of double kissing mini-culotte stenting vs. mini-culotte stenting using drug-eluting stents for the treatment of true coronary bifurcation lesions

**DOI:** 10.3389/fcvm.2024.1336750

**Published:** 2024-04-09

**Authors:** Sheng Tu, Linlin Zhang, Qingqing Tian, Fudong Hu, Ying Wang, Lianglong Chen

**Affiliations:** ^1^ Department of Cardiology, Bozhou People’s Hospital, Bozhou Hospital Affiliated to Anhui University of Science and Technology, Bozhou Hospital Affiliated to Anhui Medical University, Bozhou, Anhui, China; ^2^ Department of Cardiology, Union Hospital, Fujian Medical University, Fujian, China; ^3^ Department of Cardiology, Provincial Institute of Coronary Artery Disease, Fujian, China; ^4^ Department of Cardiology, The First Affiliated Hospital of Zhengzhou University, Zhengzhou, Henan, China; ^5^ Department of Dermatology, Bozhou People’s Hospital, Bozhou Hospital Affiliated to Anhui University of Science and Technology, Bozhou Hospital Affiliated to Anhui Medical University, Bozhou, Anhui, China

**Keywords:** percutaneous coronary intervention, coronary bifurcation lesions, culotte stenting, stent thrombosis, DK mini-culotte stenting

## Abstract

**Objective:**

This study aimed to compare the clinical outcomes of double kissing mini-culotte (DKMC) stenting with those of mini-culotte (MC) stenting in treating patients with true coronary bifurcation lesions (CBLs) in the clinical real world.

**Methods:**

This retrospective observational cohort study included 180 consecutive patients with true CBLs (Medina type 1,1,1; 1,0,1; 0,1,1). All eligible patients underwent coronary angiography and percutaneous coronary intervention with two-stent techniques in our hospital; among them, 97 received DKMC treatment and 83 MC treatment. The primary clinical endpoints were the major adverse cardiovascular events (MACE), which included cardiac death, myocardial infarction, and target vessel/lesion revascularization (TVR/TLR). The secondary endpoints were stent thrombosis, in-stent restenosis, and individual components of MACE.

**Results:**

Quantitative coronary angiography analysis (at 5 years) revealed that late lumen loss (0.25 ± 0.41 mm vs. 0.14 ± 0.32 mm, *P* = 0.032) and segmental diameter restenosis of the side branch (27.84 ± 12.34% vs. 19.23 ± 9.76%, *P* = 0.016) were lower in the DKMC treatment group than that in the MC treatment group. Notably, compared to that in the MC treatment group, the cumulative event rate of MACE at 5 years (22.8% vs. 8.3%, *P* = 0.007) and TVR/TLR (17.7% vs. 6.3%, *P* = 0.018) was higher in the DKMC treatment group, driven mainly by TVR/TLR. Especially, the DKMC group was related to a significant reduction in the primary and secondary endpoints in high-risk patients.

**Conclusion:**

DKMC treatment was associated with less late lumen loss and restenosis in the side branch and a lower rate of cumulative MACE and TVR/TLR. DKMC treatment is more effective for treating true CBLs than MC treatment; however, these findings warrant further confirmation through a randomized clinical trial.

## Introduction

Coronary bifurcation lesions (CBLs) account for about 15%–20% of lesions seen in patients requiring percutaneous coronary intervention (PCI) (1–3) and are challenging to treat. Compared with non-CBLs, CBL PCI technology is more difficult to handle, with lower success rates, higher incidences of complications, poorer long-term efficacy, and an increased risk of stent thrombosis (ST) (4–7).

PCI treatment of CBLs can be summarized as simple single-stent and complex double-stent strategies. The current expert consensus recommends CBLs with simple strategies, such as single stents or, if necessary, T stents. However, in treating CBLs with single stenting, the main vessel (MB) is undoubtedly well treated but may cause compression, dissection, acute occlusion, and even permanent loss of the side branch (SB). Therefore, the double-stent strategy, which avoids loss of intraoperative vital vascular occlusion and improves operation safety, is still an indispensable option for clinicians in treating severe true CBLs (8).

Popular used complex double-stent strategies including crush and culotte stenting. The latter is derived from several variants after several generations of improvement. The European Bifurcation Club (EBC) recommends mini-culotte (MC) stenting as a treatment approach for true CBLs (9). As a novel culotte technique, double kissing mini-culotte stenting (DKMC) has been associated with good clinical outcomes, while bench test data suggested that an additional kissing dilation approach facilitates the culotte technique (10–13). Nonetheless, no solid data confirm whether DKMC is superior to MC in treating true CBLs.

Therefore, we conducted an observational cohort study to evaluate the clinical efficacy of MC and DKMC in treating complex CBLs.

## Methods

### Study population

**Figure 1:**
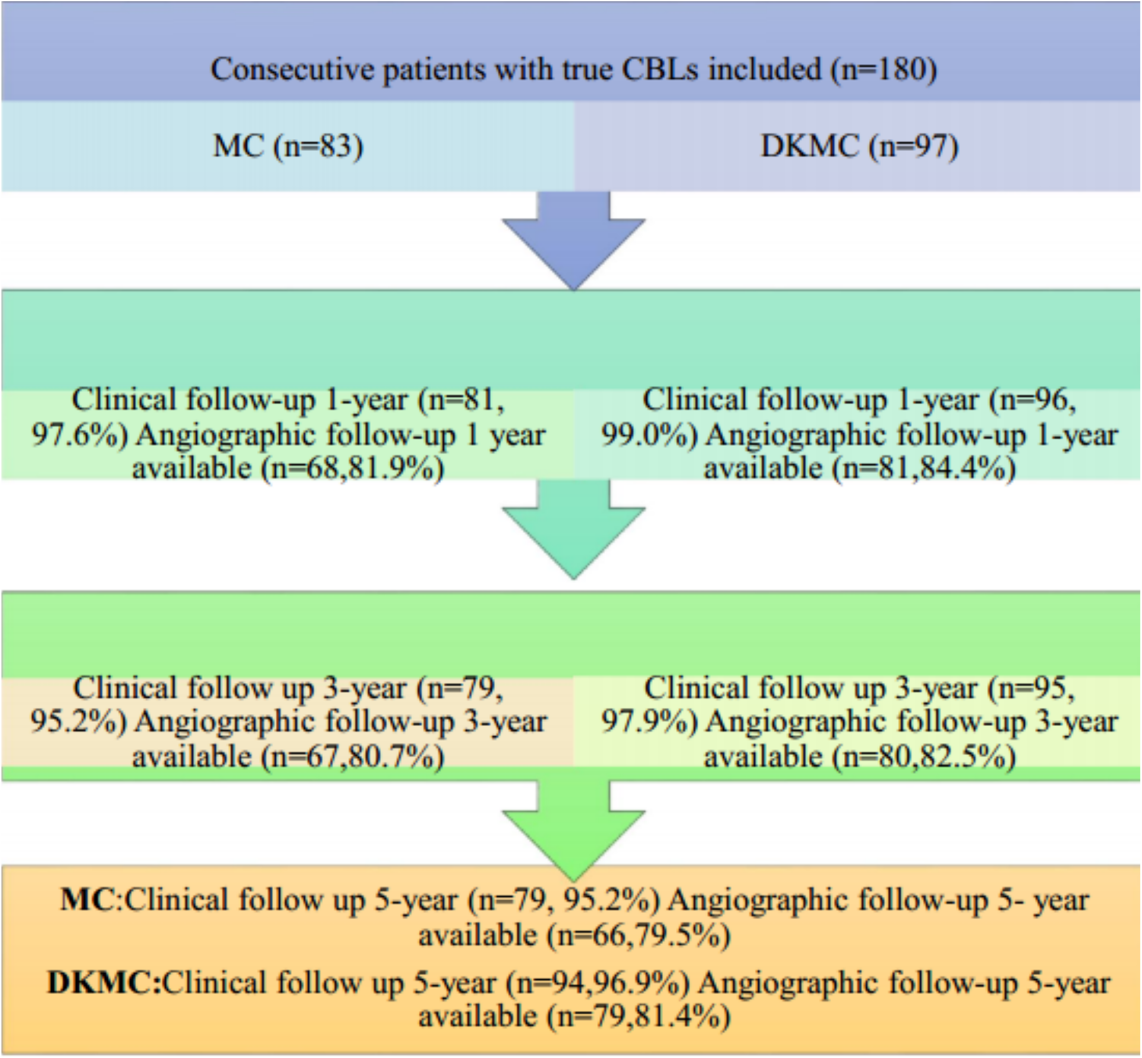
Clinical follow-up was scheduled at 1, 3, and 5 years after revascularization, and then annually thereafter. Routine angiographic follow-up was recommended at 1, 3, and 5 years after PCI.

This observational cohort study included 180 consecutive “all comer” patients with true CBLs (≥50% diameter stenosis) diagnosed by angiography from 4,562 patients who were treated with PCI in the enrollment period and no less than 60 individuals in each group according to clinical results and statistical requirements, who were treated by either MC or DKMC with drug-eluting stents (DES) between January 2010 and March 2015. Patients were treated with the upfront or rescue two-stent strategy (MC group: 70 cases of the upfront two-stent strategy and 13 cases of the rescue two-stent strategy; DKMC group: 80 cases of the upfront two-stent strategy and 17 cases of the rescue two-stent strategy). The clinical flow charts of this mini-culotte stent technique are demonstrated in Figure 1.

The study was conducted in accordance with the Declaration of Helsinki. The protocol was approved by the Medical Ethics Committee of Union Hospital, Fujian Medical University. The identity of patients remained anonymous, and the requirement for informed consent was waived due to the observational nature of the study (14, 15).

PCI was determined by an experienced heart team (interventional cardiologists) based on clinical risk, angiographic characteristics, and patients' preferences. All patients signed a written informed consent form.

The synergy between PCI with Taxus and Cardiac Surgery (SYNTAX) scores was calculated for every patient to evaluate cardiac operative risk scores.

### Procedures

All interventional procedures were performed as previously described and shown in Supplementary Material (Supplementary material S1 and Videos S1, S2) (10, 16, 17). The proximal optimizing technique (POT) was conducted in the two-stent strategy (18, 19). The stents were mainly selected from Resolute (Medtronic Cardiovascular, Santa Rosa, CA, USA), Xience V (Abbott Vascular, Santa Clara, CA, USA), Firebird-2 (Microport Co., Shanghai, China), and Excel (JW Medical System, Weihai, China). Bifurcation lesions were treated using either MC or DKMC (16). The stenting strategy was at the discretion of operators according to the lesions' characteristics and their experiences. Final kissing balloon dilation (fKBD) was performed with a non-compliant balloon according to the named time and pressure in the two-stent procedure. An intra-aortic balloon pump (IABP) was used in high-risk patients with severe heart failure. The use of intravascular ultrasound (IVUS) and the choice of a particular DES were at the discretion of the operators. Within 48 h after the procedure, cardiac troponin (cTn) values above 5 times the 99th percentile upper reference limit (URL) after PCI or 10 times after coronary artery bypass grafting (CABG) were used to define periprocedural PCI or CABG-related MI in patients with normal baseline cTn levels. “+” graft occlusion was defined according to the Academic Research Consortium (ARC)-like definition (for CABG). No patients undergoing CABG were enrolled.

All patients undergoing PCI were administered a loading dose of aspirin (300 mg) plus clopidogrel (300 or 600 mg) or ticagrelor (180 mg) before or during intervention procedures. Periprocedural anticoagulation followed the standard treatment.Patients received unfractionated heparin 100 IU/kg intravenously during the procedure, which was corrected to maintain an activated clotting time >300 s. Whether to use glycoprotein IIb/IIIa receptor inhibitors was left to the discretion of the operators. After discharge, all patients treated with PCI were prescribed a standard dual antiplatelet therapy regimen (aspirin 100 mg daily and clopidogrel 75 mg daily or ticagrelor 90 mg twice daily) for at least 12 months and were indefinitely continued with aspirin.

Other post-procedure medication treatments such as statins, angiotensin-converting enzyme inhibitors (ACEIs) or angiotensin receptor blockers (ARBs), and beta-blockers were prescribed to patients undergoing PCI according to current clinical practice.

### Follow-up

Clinical follow-up was scheduled at 1, 6, and 12 months (1 year), 3 years, and 5 years after revascularization, followed by annual visits thereafter. Routine angiographic follow-up was recommended at 1, 3, and 5 years after PCI. Angiography was performed beforehand if symptoms of angina recurred. Participants who did not adhere to the recommended follow-up processes were interviewed via telephone.

### Study endpoints and definitions

The primary endpoint of this study was a patient-oriented composite of major adverse cardiac and cerebrovascular events (MACE) at 1-, 3-, and 5-year follow-ups, which included all-cause death, myocardial infarction (MI), and target vessel revascularization (TVR) or target lesion revascularization (TLR). In the analysis of cumulative endpoints, events were counted only once, whichever occurred first.

The secondary endpoints were a composite safety endpoint of all-cause death/MI/stroke, individual components of MACE, and ST.

Death was defined as post-procedure death from any cause and was classified as from either cardiac or non-cardiac causes, according to the ARC definition (20). Death was considered as cardiac origin unless a non-cardiac origin has definitely been proved. Cardiac death was defined as any death due to a cardiac cause (e.g., MI, low-output heart failure, fatal arrhythmias), procedure-related death, or death of unknown cause.

MI was defined according to the third universal definition of myocardial infarction (21). Evidence for MI mainly included elevated cTn with at least one value >99th percentile URL, symptoms of myocardial ischemia, electrocardiographic changes, and angiographic characteristics. Within 48 h after the procedure, cTn values >5 times the 99th percentile URL after PCI or 10 times after CABG were used to define periprocedural PCI or CABG-related MI in patients with normal baseline cTn levels (≤99th percentile URL). If the baseline values were elevated, stable, or falling, a rise of cTn values >20% was also considered evidence of periprocedural PCI-related MI. Q-wave MI was defined as MI together with a new pathologic Q-wave in no less than two contiguous leads after index treatment.

TVR was defined as any surgical or percutaneous repeat revascularization of any segment of the stented vessel (target lesion, upstream or downstream branches) within 1 year, including the left main, left anterior descending, and left circumflex coronary arteries. A planned staged PCI was not considered a TVR.

The occurrence of definite ST was defined according to the ARC definition (for PCI), and graft occlusion was defined according to the ARC-like definition (for CABG) (22, 23).

### Statistical analysis

Continuous variables were presented as mean ± SD, and categorical variables were presented as number (%). Comparisons between the MC and DKMC groups in baseline characteristics were performed by the *t*-test or Wilcoxon rank-sum test for continuous data and using the chi-squared test or Fisher's exact test for categorical data, as appropriate. Cumulative event curves of the MC and DKMC groups were constructed by the Kaplan–Meier method and were compared using the log-rank test.

A *P*-value <0.05 was considered statistically significant, and all tests were two-tailed. All statistical analyses were performed with SPSS software (version 22.0, SPSS, IBM Corporation, Armonk, New York, USA).

## Results

### Baseline characteristics

A total of 180 consecutive patients with true CBLs who received MC (*n* = 83) or DKMC (*n* = 97) treatment between January 2010 and March 2015 were included in the study. All these patients were completely followed up for 5 years (Figure 1). Baseline clinical characteristics, angiographic characteristics, and cardiac operative risk scores are given in Tables 1–3. The two groups exhibited no significant differences in other clinical characteristics, angiographic characteristics, cardiac operative risk scores, and procedural characteristics (all *P*’s > 0.05).

**Table 1:** Baseline clinical characteristics.

Variables	MC (*n* = 83)	DKMC (*n* = 97)	*P*-value
Age, years	63.28 ± 10.11	64.61 ± 9.35	0.906
<65	53 (63.9)	59 (60.8)	0.676
≥65	30 (36.1)	38 (39.1)	
Gender, *n* (%)			0.674
Male	72 (86.7)	82 (84.6)	
Female	11 (13.3)	15 (15.5)	
Hypertension, *n* (%), mmHg	51 (61.4%)	62 (63.9%)	0.732
Systolic BP	135.1 ± 15.4	130.6 ± 19.3	0.326
Diastolic BP	78.2 ± 13.3	75.6 ± 11.4	0.565
Diabetes	20 (24.1)	22 (22.7)	0.823
Fasting glucose, mmol/L	5.62 ± 1.43	6.12 ± 1.98	0.335
Insulin-dependent, *n* (%)	4 (20.0)	5 (22.7)	0.830
Hyperlipidemia, *n* (%)	25 (30.1)	38 (39.2)	0.204
TC, mmol/L	3.98 ± 1.16	4.16 ± 1.21	0.568
LDL-C, mmol/L	2.41 ± 1.22	2.67 ± 1.03	0.431
Smoking, drinking, *n* (%)			
Current smoking	31 (37.3)	33 (34.0)	0.642
Quit smoking	8 (9.6)	12 (12.4)	0.561
Current drinking	36 (43.4)	32 (33.0)	0.168
Quit drinking	13 (15.7)	16 (16.5)	0.880
Peripheral vascular disease, *n* (%)	4 (4.8)	5 (5.2)	0.918
Serum creatinine, μmol/L	85.8 ± 20.6	87.4 ± 24.3	0.874
Previous MI, *n* (%)	12 (14.5)	18 (18.6)	0.462
Previous PCI, *n* (%)	15 (18.1)	20 (20.6)	0.667
LVEF, %	61.4 ± 10.2	63.6 ± 13.0	0.386
<50%, *n* (%)	14 (16.9)	15 (15.5)	0.798
Clinical indication			
Stable angina pectoris, *n* (%)	9 (10.8)	14 (14.4)	0.472
Unstable angina, *n* (%)	51 (61.4)	63 (64.9)	0.627
Non-STEMI	13 (15.7)	11 (11.3)	0.395
STEMI	10 (12.0)	8 (8.2)	0.397

BP, blood pressure; TC, total cholesterol; LDL-C, low-density lipoprotein cholesterol; LVEF, left ventricular ejection fraction; STEMI, ST-segment elevation myocardial infarction.

Values are mean ± SD or *n* (%).

**Table 2:** Lesion characteristics and procedural risk scores.

Variables	MC (*n* = 83)	DKMC (*n* = 97)	*P*-value
Lesion location			
LM/LAD/LCX, *n* (%)	26 (31.3)	20 (22.7)	0.101
LAD/DB, *n* (%)	45 (54.2)	59 (60.8)	0.371
LCX/OM, *n* (%)	6 (7.2)	14 (14.4)	0.125
RCA/PDA/PLA	6 (7.2)	3 (3.1)	0.204
Lesion type			
Medina 1,1,1, *n* (%)	60 (72.3)	62 (63.9)	0.231
Medina 1,0,1, *n* (%)	6 (7.2)	5 (5.2)	0.562
Medina 0,1,1, *n* (%)	17 (20.5)	30 (30.9)	0.112
Plaque features			
B2/C-type lesion, *n* (%)	62 (74.7)	78 (80.4)	0.358
Total occlusion, *n* (%)	11 (13.3)	7 (7.2)	0.100
Distortion or calcification, *n* (%)	10 (12.0)	12 (12.4)	0.947
SYNTAX score, points	21.3 ± 6.4	22.6 ± 6.8	0.938

LAD, left anterior descending; LCX, left circumflex; LM, left main; DB, diagonal branch; OM, oblique marginal; PDA, posterior descending artery; PLA, posterior artery of left ventricle; RCA, right coronary artery.

Values are *n* (%) or mean ± SD.

**Table 3:** Procedural characteristics.

Variables	MC (*n* = 83)	DKMC (*n* = 97)	*P*-value
Radial access, *n* (%)	53 (63.9)	67 (69.1)	0.459
Arterial sheath, F	6.77 ± 0.42	6.89 ± 0.61	0.865
Pre-dilation			
Main vessel, *n* (%)	64 (77.1)	69 (71.1)	0.363
Side branch, *n* (%)	49 (59.0)	51 (52.6)	0.385
Main vessel stent			
Number	1.37 ± 0.52	1.48 ± 0.58	0.751
Maximum diameter, mm	3.69 ± 0.37	3.82 ± 0.40	0.198
Total length, mm	35.2 ± 17.3	33.5 ± 20.5	0.517
Side branch stent			
Number	1.03 ± 0.23	1.05 ± 0.20	0.852
Maximum diameter, mm	3.37 ± 0.38	3.41 ± 0.44	0.579
Total length, mm	24.1 ± 11.6	21.5 ± 10.9	0.626
Final kissing balloon dilatation, *n* (%)	74 (89.2)	97 (100)	0.010
Double kissing balloon dilatation, *n* (%)	0 (0)	100 (100)	0.000
Stents type, *n* (%)			
SES	76 (91.5)	88 (90.7)	0.843
ZES	5 (6.0)	5 (5.2)	0.800
EES	2 (2.5)	4 (4.1)	0.523
Fluoroscopy time, min	81.5 ± 18.9	78.1 ± 23.6	0.753
Contrast volume, ml	338.2 ± 100.6	311.5 ± 106.3	0.657
IABP support, *n* (%)	3 (3.6)	5 (5.2)	0.617
IVUS assessment, *n* (%)	19 (22.9)	32 (33.0)	0.134

SES, sirolimus-eluting stent, ZES, zotarolimus-eluting stent, EES, everolimus-eluting stent.

Values are *n* (%) or mean ± SD or number (%).

### Clinical outcomes

#### Overall population

Of the 180 enrolled patients, 177 were followed up during 1 year, including 81 patients in the MC group (97.6% of the clinical follow-up) and 96 patients in the DKMC group (99.0% of the clinical follow-up). Quantitative coronary angiography (QCA) measurements of the two groups [68 patients (84.0%) in the MC treatment group and 81 patients (84.4%) in the DKMC treatment group] were followed up within 1 year after operation. At 3 years, the clinical follow-up was completed in 80 (96.4%) patients in the MC treatment group and 96 (99.0%) patients in the DKMC treatment group and the QCA follow-up was completed in 67 (80.7%) patients in the MC treatment group and 80 (82.5%) patients in the DKMC treatment group. At 5 years, the clinical follow-up was completed in 79 (95.2%) patients in the MC treatment group and 96 (99.0%) patients in the DKMC treatment group and the QCA follow-up was completed in 66 (79.5%) patients in the MC treatment group and 79 (81.4%) patients in the DKMC treatment group.

At 1 year, QCA analysis showed (Table 4) that late lumen loss (0.12 ± 0.42 mm vs. 0.22 ± 0.39 mm, *P* = 0.045) and segmental diameter restenosis of the side branch (17.56 ± 10.23% vs. 23.62 ± 13.28%, *P* = 0.032) were lower than in the MC group. There were similar rates of binary restenosis in the parent main vessel (1.2% vs. 2.9%) and the main branch (4.8% vs. 5.9%) in both groups, with a numerically lower rate of binary restenosis in the side branch in the DKMC group (6.2% vs. 13.2%, *P* = 0.141) irrespective of no significant differences.

**Table 4:** QCA measurements immediately and at index 1-, 3-, and 5-year follow-ups.

	*N* MC/DKMC	PMV			MV			SB		
		MC	DKMC	*P*	MC	DKMC	*P*	MC	DKMC	*P*
Lesion length		7.65 ± 4.08	7.78 ± 4.14	0.862	14.93 ± 9.48	14.86 ± 7.65	0.876	14.35 ± 8.92	12.24 ± 9.12	0.818
RVD										
Prior stenting	83/97	3.49 ± 0.39	3.47 ± 0.47	0.801	3.01 ± 0.37	3.03 ± 0.41	0.812	2.48 ± 0.36	2.53 ± 0.42	0.672
After PCI	83/97	3.67 ± 0.43	3.64 ± 0.51	0.715	3.18 ± 0.39	3.19 ± 0.43	0.765	2.71 ± 0.37	2.80 ± 0.39	0.264
1-year follow-up	68/81	3.56 ± 0.45	3.52 ± 0.51	0.614	3.07 ± 0.33	3.11 ± 0.39	0.586	2.59 ± 0.38	2.71 ± 0.41	0.442
3-year follow-up	67/80	3.53 ± 0.55	3.51 ± 0.48	0.687	3.06 ± 0.28	3.10 ± 0.36	0.612	2.58 ± 0.34	2.69 ± 0.39	0.508
5-year follow-up	66/79	3.50 ± 0.57	3.51 ± 0.43	0.879	3.06 ± 0.29	3.10 ± 0.33	0.636	2.55 ± 0.32	2.66 ± 0.35	0.612
MLD/MSD										
Prior stenting	83/97	2.19 ± 0.57	2.07 ± 0.54	0.459	2.11 ± 0.52	2.14 ± 0.47	0.768	2.01 ± 0.49	2.04 ± 0.43	0.436
After PCI	83/97	3.24 ± 0.47	3.25 ± 0.45	0.586	2.65 ± 0.41	2.85 ± 0.44	0.505	2.26 ± 0.36	2.44 ± 0.38	**0**.**001**
1-year follow-up	68/81	3.21 ± 0.41	3.23 ± 0.43	0.612	2.62 ± 0.52	2.81 ± 0.59	0.726	2.12 ± 0.32	2.41 ± 0.41	**0**.**021**
3-year follow-up	67/80	3.20 ± 0.42	3.22 ± 0.39	0.606	2.59 ± 0.48	2.78 ± 0.45	0.689	3.18 ± 0.29	2.69 ± 0.39	**0**.**016**
5-year follow-up	66/79	3.18 ± 0.39	3.20 ± 0.33	0.755	2.57 ± 0.47	2.75 ± 0.43	0.752	4.24 ± 0.36	2.77 ± 0.35	**0**.**009**
Diameter stenosis (%)										
Prior stenting	83/97	85.6 ± 10.41	83.6 ± 9.71	0.618	82.91 ± 11.2	83.62 ± 9.8	0.186	80.42 ± 9.81	78.62 ± 10.71	0.368
After PCI	83/97	8.41 ± 3.25	8.16 ± 3.08	0.524	8.02 ± 3.19	7.72 ± 3.41	0.753	21.79 ± 10.56	14.58 ± 6.84	**0**.**000**
1-year follow-up	68/81	8.33 ± 5.46	8.24 ± 7.15	0.684	14.66 ± 9.56	11.36 ± 11.12	0.428	23.62 ± 13.28	17.56 ± 10.23	**0**.**032**
3-year follow-up	67/80	8.31 ± 4.41	8.23 ± 7.15	0.697	16.25 ± 8.39	13.27 ± 10.06	0.561	26.78 ± 11.26	18.32 ± 10.18	**0**.**028**
5-year follow-up	66/79	8.30 ± 3.46	8.22 ± 7.15	0.736	17.31 ± 7.36	15.16 ± 9.72	0.623	27.84 ± 12.34	19.23 ± 9.76	**0**.**016**
LLL, mm										
1-year follow-up	68/81	0.11 ± 0.42	0.09 ± 0.45	0.753	0.22 ± 0.29	0.15 ± 0.38	0.548	0.22 ± 0.39	0.12 ± 0.42	**0**.**045**
3-year follow-up	67/80	0.13 ± 0.38	0.13 ± 0.53	0.802	0.24 ± 0.19	0.17 ± 0.34	0.642	0.24 ± 0.43	0.14 ± 0.41	**0**.**042**
5-year follow-up	66/79	0.14 ± 0.32	0.14 ± 0.42	0.846	0.25 ± 0.17	0.18 ± 0.28	0.726	0.25 ± 0.41	0.14 ± 0.32	**0**.**035**
Accumulative restenosis, *n* (%)										
1-year follow-up	68/81	2 (2.94)	1 (1.47)	0.878	4 (5.88)	4 (4.94)	0.912	9 (13.24)	5 (6.17)	0.125
3-year follow-up	67/80	2 (2.50)	1 (1.25)	0.877	5 (7.46)	4 (5.00)	0.915	11 (16.42)	6 (7.50)	0.092
5-year follow-up	66/79	3 (4.54)	2 (2.53)	0.873	5 (7.58)	4 (5.06)	0.780	13 (19.70)	6 (7.59)	**0**.**032**

MLD/MSD, minimal luminal diameter before stenting/minimal stent diameter after stented; RVD, reference vessel diameter; LLL, late lumin loss.

Values are mean ± SD or *n* (%). Restenosis was defined as ≥50% diameter stenosis at index 1-, 3-, and 5-year follow-ups.

Bold values are statistically significant (*P *< 0.05).

**Figure 2:**
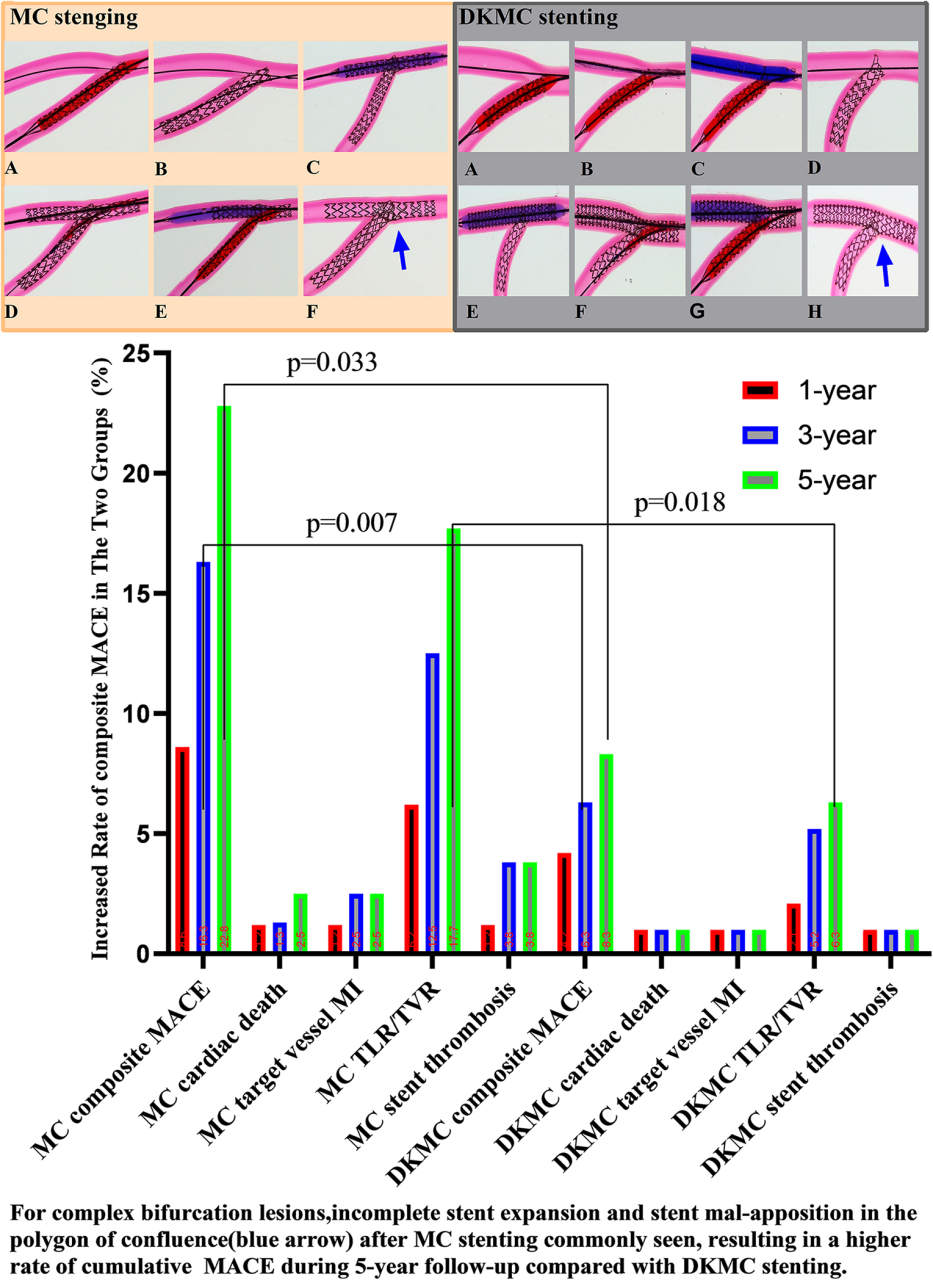
Top left: images of stents deployed in mock phantoms using the MC strategy. The detailed MC strategy process is shown in Supplementary Videos S1, S2 and pre-publication literature in Supplement material S1. In the culotte technique, the first stent is often deployed in the SB, usually across the SB to the main vessel (MV), with a protrusion about 3–5 mm (**A, B**). The MV then needs to be rewired through the strut of the first stent, and after predilatation with a non-compliant balloon (**C**), a second stent is then implanted (**D**). The procedure is completed after rewiring the SB by final kissing balloon dilatation of both branches (**E**). Then, with the proximal optimize technique (POT), a suitable non-compliance balloon is used to the proximal MB stent as necessary to end the procedure (**F**). Top right: images of stents deployed in mock phantoms using the DKMC strategy. The detailed DKMC strategy process is shown in Supplementary Videos S2 and pre-publication literature in Supplementary Appendix 1. DKMC strategy: Preimbed a balloon in the MB and position the side branch (SB) stent with mini-protrusion (1-2 mm) into the MV. The wire was then retrieved to the bifurcation and gently advanced through the struts of the stents (**A**), which was subsequently prodilated with a 1.5-mm balloon (if necessary in real world) (**B**); then with the NC-balloon in MB (match diameter of MB) and the NC-balloon ( match diameter of SB) for the first sequential intermediate kissing balloon dilation (siKBD) with 10 atmospheric pressure (ATM) (**C**), withdraw the two balloons and SB wire and insert the MB stent. After deploying the MB stent (**D,E**), withdraw the stent balloon. The wire was then retrieved to the SB and gently advanced through the struts (**F**), with a 1.5-mm balloon (if necessary) used for prodilation, followed by the final kissing balloon dilation (fKBD) procedure (**G**). The procedure concludes with POT (**H**). At 1-year follow-up, 2 (1.1%) patients suffered cardiac death, 2 (1.1%) patients suffered definite stent thrombosis, 7 (4.0%) patients received TVR, and cumulative MACE occurred in a total of 11 (6.2%) patients. At 3- and 5-year follow-ups, the accumulative incidences of MACE were 16.3% and 22.8% in the MC stenting group and 6.3% and 8.3% in the DKMC group, respectively (*P* < 0.05) (Table 5), mainly resulting from they increased rates of TLR/TVR (12.5% vs. 5.2%, *P* = 0.085; 17.7% vs. 6.3%, *P* = 0.018) in the MC stenting group.

At the 1-year follow-up, 2 (1.1%) patients suffered cardiac death, 2 (1.1%) patients suffered definite stent thrombosis, 7 (4.0%) patients received TVR and cumulative MACE occurred in 11 (6.2%) patients. At 3- and 5-year follow-ups, the accumulative incidences of MACE were 16.3% and 22.8% in the MC stenting group and 6.3% and 8.3% in the DKMC group, respectively (*P* < 0.05) (Table 5), mainly resulting from increased rates of TLR/TVR (12.5% vs. 5.2%; *P* = 0.085; 17.7% vs. 6.3%; *P* = 0.018) in the MC stenting group. The detailed cumulative clinical outcomes of the two groups are presented in Table 5 and Figure 2.

**Table 5:** Clinical outcomes at follow-up (total population).

Outcome	MC	DKMC	*P*-value
1-year follow-up	(*n* = 81)	(*n* = 96)	* *
Accumulative composite MACE	7 (8.6)	4 (4.2)	0.219
Cardiac death, *n* (%)	1 (1.2)	1 (1.0)	0.904
Target vessel MI, *n* (%)	1 (1.2)	1 (1.0)	0.904
TLR/TVR, *n* (%)	5 (6.2)	2 (2.1)	0.164
Stent thrombosis, *n* (%)	1 (1.2)	1 (1.0)	0.408
3-year follow-up	(*n* = 80)	(*n* = 96)	
Accumulative composite MACE	13 (16.3)	6 (6.3)	**0**.**033**
Cardiac death, *n* (%)	1 (1.3)	1 (1.0)	0.897
Target vessel MI, *n* (%)	2 (2.5)	1 (1.0)	0.457
TLR/TVR, *n* (%)	10 (12.5)	5 (5.2)	0.085
Stent thrombosis, *n* (%)	3 (3.8)	1 (1.0)	0.230
5-year follow-up	(*n* = 79)	(*n* = 96)	
Accumulative composite MACE	18 (22.8)	8 (8.3)	**0**.**007**
Cardiac death, *n* (%)	2 (2.5)	1 (1.0)	0.882
Target vessel MI, *n* (%)	2 (2.5)	1 (1.0)	0.882
TLR/TVR, *n* (%)	14 (17.7)	6 (6.3)	**0**.**018**
Stent thrombosis, *n* (%)	3 (3.8)	1 (1.0)	0.225

STEMI, ST-segment elevation myocardial infarction.

Values are *n* (%) unless otherwise indicated. Outcome rates are Kaplan–Meier estimates with *P*-values from the log-rank test, unadjusted.

Bold values are statistically significant (*P *< 0.05).

## Discussion

These results suggest that DKMC and MC stenting techniques are safe and effective in treating complex CBLs. The incidence of MACE at the 1-year follow-up was similar in both groups, but DKMC was associated with less late lumen loss and restenosis in the side branch, with a numerically lower rate of cumulative MACE and TVR/TLR. In addition, MC stenting for CBLs was associated with significantly increased rates of accumulative incidences of MACE over 5 years of follow-up. DKMC stenting is more effective in treating true CBLs than MC stenting.

Expert consensus has suggested that provisional T-stenting is superior to systematic double-stent techniques and should be used as the initial treatment strategy for most CBLs (class I recommendations). The single-stent strategy is mainly used for SB with mild-to-moderate localized stenosis. A double-stent strategy is conducted for large SB with a high risk of occlusion. In such cases, MB is stented while the ostium of SB is squeezed to occlusion, after which SB (IIA class) can be implanted. Clinically, the biggest technical challenge of remedial implantation of SB stents is the precise positioning of the stent in the SB ostium. Therefore, current recommendations for EBC as a provisional strategy are considered the standard of care where two stenting techniques are reserved for unsuccessful side branch results. Therefore, MC can be used to further save the side branch vessels and for re-revascularization. As DKMC stenting is associated with good clinical outcomes, we evaluated the MC and DKMC stenting bifurcation techniques in this retrospective clinical practice.

Based on the results of several recently randomized clinical trials, there was no sufficient evidence to support an absolute advantage in the clinical efficacy of many CBLs (4, 11, 24). In the EBC TWO study, 200 patients with CBL and significant stenosis were randomized to either a provisional T-stenting or a culotte stenting group. In the provisional group, 60% received T-stenting. At 12 months, the MACE rate was 7.7% for the provisional group and 10.3% for the culotte group (25). Zhang et al. compared provisional stenting (PS) with culotte stenting and found equal MACE rates at 9 months (26). The BBK II trial, involving 300 patients randomized to T-stenting and small protrusion technique (TAP) stenting or culotte stenting, reported restenosis rates of 17% for TAP and 6.5% for culotte (*P* = 0.006), with 1-year TLR rates of 12.0% and 6.0%, respectively (*P* = 0.069) (27). Culotte stenting showed lower angiographic restenosis than TAP. Regarding the DK mini-culotte technique vs. T-provisional stenting, Fan et al. reported lower MACE rates (4.55% vs. 13.6%) and TLR/TVR rates (1.52% vs. 12.12%, *P* = 0.033) in the DK mini-culotte group. SB restenosis was also lower in the DK mini-culotte group (5.6% vs. 22.4%, *P* = 0.014) (10). However, this study's results, which were based on complex coronary bifurcation lesions while the standard MC stenting was used as a control, suggested that DKMC's technical improvements are successful and effective.

The main difference between MC and DKMC is that DKMC is based on MC and increases the sequential intermediate balloon kissing (siKBD), so the difference between MC and DKMC is likely due to siKBD. Bench test and intravascular ultrasound findings reported that a “napkin,” gap, or metallic ridge is usually seen at the ostial SB after classic culotte stenting, leading to incomplete coverage of the ostial SB and resulting in increased TLR. While MC stenting originates from classic culotte stenting and features fewer struts of SB stretching into the MB lumen, there are still inherent shortcomings associated with MC (28). In addition, clinic trials revealed that an increased number of connectors improve device-related outcomes among a range of contemporary very thin stent models. In contrast, DKMC introduces one time of kissing balloon inflation (siKBD) before stenting the MB. Finishing the procedure with a final kissing balloon inflation enables the full coverage of the ostial SB and reduces restenosis in the ostial SB, which may result in less TVR/TLR (29).

Although antiplatelet therapy was left at the physician's discretion, patients with acute coronary syndrome were enrolled. The duration of DAPT may be associated with different outcomes according to admission diagnosis. Even in complex PCI scenarios such as bifurcation lesions, an extended DAPT strategy was associated with reducing MACE in acute coronary syndromes (ACS) in this cohort. Although there were no convincing data after stenting bifurcation lesions, a complex stenting approach resulted as an independent factor for TVR/TLR. A similar TVR rate (6.3%) after DKMC stenting in the present study was slightly lower than the DK-Crush III previously reported (30). It is possible that both DK crush and DKMC techniques are safe for true CBLs and that optimizing the expansion of the SB stent, as done with the DK crush technique, could lead to a lower risk of TVR/TLR. In addition, in the present study, restenotic lesions were most commonly localized in the SB, which is consistent with the previous results. Thus, we argue that DKMC stenting was superior to MC stenting in the current study.

## Limitations

There are several limitations in the present study. First, this was an observational single-center study with a relatively small sample size. Moreover, the 5-year follow-up was inadequate to compare the treatment outcomes after PCI with MC vs. DKMC; thus, the durability of these results on a long-term basis remains unknown.

## Conclusion

Our data suggested that DKMC stenting for CBLs medical treatment is associated with less late lumen loss and restenosis in the side branch, with a numerically lower cumulative MACE and TVR/TLR than standard MC stenting; yet these findings need to be further confirmed by a randomized clinical trial.

## Data Availability

The original contributions presented in the study are included in the article/**Supplementary Material**, further inquiries can be directed to the corresponding author.
