# Supplementary material for: Five-year outcomes of double kissing mini-culotte stenting vs. mini-culotte stenting using drug-eluting stents for the treatment of true coronary bifurcation lesions
Source: Front Cardiovasc Med. 2024 Apr 9;11:1336750. doi: 10.3389/fcvm.2024.1336750 (PMC11035737; doi:10.3389/fcvm.2024.1336750)
Supplement: Supplementary file 3 [file Datasheet1.pdf]

EuroIntervention 2019;15:465-472 published online December 2018

Double kissing mini-culotte versus mini-culotte stenting: insights from micro-computed tomographic imaging of bench testing

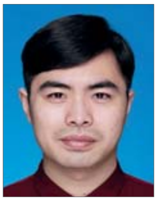

Fudong Hu[1,2], MD, PhD; Sheng Tu[1], MD, PhD; Wei Cai[1], MD, PhD; Zhengming Jiang[2], MD; Hong Zheng[1], MD; Liyan Xiao[1], MD; Chunyan Qiu[1], MD; Chang Xiong[1], MD; Yaner Yao[1], MD; Lianglong Chen[1]*, MD, PhD, FACC

1. Department of Cardiology, Union Hospital, Fujian Medical University, Fuzhou, China; 2. Department of Cardiology, The First Affiliated Hospital of Zhengzhou University, Zhengzhou, China

This paper also includes supplementary data published online at: https://eurointervention.pcronline.com/doi/10.4244/EIJ-D-18-00688

KEYWORDS

- balloon inflation
- bifurcation
- drug-eluting stent
- stent enhancement

Abstract

Aims: This study aimed to evaluate the morphologic characteristics of double kissing (DK) mini-culotte and mini-culotte stenting through imaging of bench testing.

Methods and results: DK mini-culotte and mini-culotte stenting were performed in two silicone bifurcated phantoms with branch vessel diameter differences of 0.5 mm (Model 1) and 1.25 mm (Model 2), and their morphologic characteristics were evaluated by micro-computed tomography. In Model 1, metal carina length (0.25±0.13 mm vs 0.55±0.15 mm), area stenosis of the side branch ostium (SBO) (4.65±3.24% vs 12.5±3.93%), and maximum distance of malapposed struts for the wall facing the SBO (0.27±0.08 mm vs 0.49±0.15 mm) were lower in the DK mini-culotte group than in the mini-culotte group. In Model 2, metal carina length (0.21±0.47 mm vs 0.47±0.12 mm), SBO area stenosis (5.13±3.37% vs 15.00±6.18%), and maximum distance of malapposed struts (0.32±0.13 mm vs 0.68±0.10 mm) were also lower in the DK mini-culotte group. The results of factorial analysis showed that maximum distance of malapposed struts tended to be shorter in Model 1 (F=4.226, p=0.062).

Conclusions: Compared with mini-culotte stenting, DK mini-culotte stenting was associated with shorter metal carina length, shorter maximum distance of malapposed struts, and smaller SBO area stenosis. Thus, DK mini-culotte stenting may obtain better morphologic characteristics.

**Corresponding author: Department of Cardiology, Union Hospital, Fujian Medical University, 29 Xin-Quan Road, Fuzhou, 350001 Fujian, P.R. China. E-mail: lianglongchenxh@163.com*

© Europa Digital & Publishing 2019. All rights reserved.

SUBMITTED ON 03/07/2018 - REVISION RECEIVED ON 1st 30/10/2018 / 2nd 28/11/2018 - ACCEPTED ON 05/12/2018

DOI: 10.4244/EIJ-D-18-00688

Abbreviations

**DK**	double kissing
**DMV**	distal main vessel
**micro-CT**	micro-computed tomography
**MLA**	minimum lumen area
**MLD**	minimum lumen diameter
**PMV**	proximal main vessel
**RLA**	reference lumen area
**RLD**	reference lumen diameter
**SBO**	side branch ostium

Introduction

Coronary bifurcation lesions are an important aspect of coronary artery disease and account for about 15-20% of percutaneous coronary intervention (PCI) in the treatment of coronary artery disease. Moreover, PCI for bifurcation lesions is still considered a complex and challenging procedure[1,2]. Selecting the appropriate one-stent or two-stent technique is very important for the treatment of bifurcation lesions. When dealing with bifurcation lesions, current guidelines and expert consensuses recommend provisional stenting and this stenting can usually end up with a two-stent technique[2,3]. However, when we encounter a large side branch (≥2.75 mm) with a significant long ostial lesion (>5 mm), an important side branch with anticipated difficulty in accessing after main branch stenting, or true left main bifurcation lesions, up-front two-stent strategies should be considered; sometimes, the side branch stent should be implanted first to avoid loss of the side branch[3].

Culotte stenting is one of the commonly used two-stent techniques, which has been shown to have acceptable clinical outcomes[4]. In the Nordic study, culotte stenting was associated with significantly better clinical results than crush stenting for the treatment of left main bifurcation lesions[5]. However, in the DKCRUSH-III study[6], the incidence rates of both major adverse cardiovascular events and side branch restenosis after DK-crush stenting were lower than those in classic culotte stenting. Therefore, innovation and improvement in correcting the technical defects of classic culotte stenting are necessary.

Recently, we proposed modified mini-culotte stenting (mini-culotte stenting) and DK mini-culotte stenting as new stenting techniques[7,8]. Although the results of clinical studies have shown that these two stenting methods could bring good clinical outcomes[7,8], the morphological effects of DK balloon dilatation in the mini-culotte stenting technique are still unclear.

In this study, we constructed vascular models of left main bifurcations, conducted bench testing of DK mini-culotte and mini-culotte stenting in the above bifurcation models *in vitro*, and then used high-resolution micro-computed tomography (micro-CT) to evaluate the morphological characteristics of these two stenting techniques.

Materials and methods

BIFURCATION PHANTOM MODELS

Coronary bifurcation models with hyperelastic properties and allowed overexpansion were made from medical silicone (Patent No. CN 203480749 U). The inner surfaces of these models had no lesions and were smooth. Left main bifurcation models with 70º distal bifurcation angles were used in this study; their internal diameters were about 4.0 mm in the main branch, and 3.5 mm (Model 1) or 2.75 mm (Model 2) in the side branch.

STENTING PROCEDURES

We used open-loop drug-eluting stents (Firebird2™; MicroPort, Shanghai, China) with diameters of 4.0 mm, 3.5 mm and 2.75 mm, which were matched with the bifurcation phantom models. DK mini-culotte and mini-culotte stenting (single-kissing balloon dilatation) were performed in two silicone bifurcation models with side branch diameter differences of 0.5 mm (Model 1) and 1.25 mm (Model 2). The operations were carried out in an aqueous bath at 37ºC, and air bubbles in and around the models were excluded. A high-resolution digital video recorder (L-12; Elmo Co., Beijing, China) was used to record the procedures. The main procedures of these two stenting techniques *in vitro* are shown in **Supplementary Figure 1** and **Supplementary Figure 2**.

MICRO-CT IMAGING

The bifurcation models of DK mini-culotte and mini-culotte stenting were scanned by a high-resolution (18 µm/pix) micro-CT device (SkyScan 1176; SkyScan, Aartselaar, Belgium), and the images were acquired at 65 kV and 385 µA. Three-dimensional images were reconstructed from the acquired cross-section images by the NRecon software (SkyScan). Image-processing operations such as rotation, electronic dissection, and measuring quantitative parameters were conducted by proprietary software (Dataview, CTVox and CTan; SkyScan).

ANALYSIS METHODS OF BIFURCATION MODELS

As shown in **Figure 1**, the two-dimensional radiographic image of each bifurcation stenting model was divided into four distinct segments: proximal main vessel (PMV), distal main vessel (DMV), side branch, and polygon of confluence. Polygon of confluence was the confluence site of the PMV and two branch vessels, the intersection of the two branch vessels formed the carina, and the distal angle between DMV and side branch named the distal bifurcation angle[9,10]. As the vessel walls were eliminated in the micro-CT imaging analysis, actual measurements were performed only for the stent images.

MEASUREMENT INDICES

The main measurement indices included reference lumen diameter (RLD), minimum lumen diameter (MLD), reference lumen area (RLA), minimum lumen area (MLA), metal carina length, side branch ostium (SBO) total area, SBO free of strut coverage area, and maximum distance of malapposed struts for the wall facing the SBO[11]. The measurement methods are shown in **Figure 1** and **Figure 2**. The proportions of diameter stenosis (%) and area stenosis (%) were calculated by (1–MLD/RLD)×100%

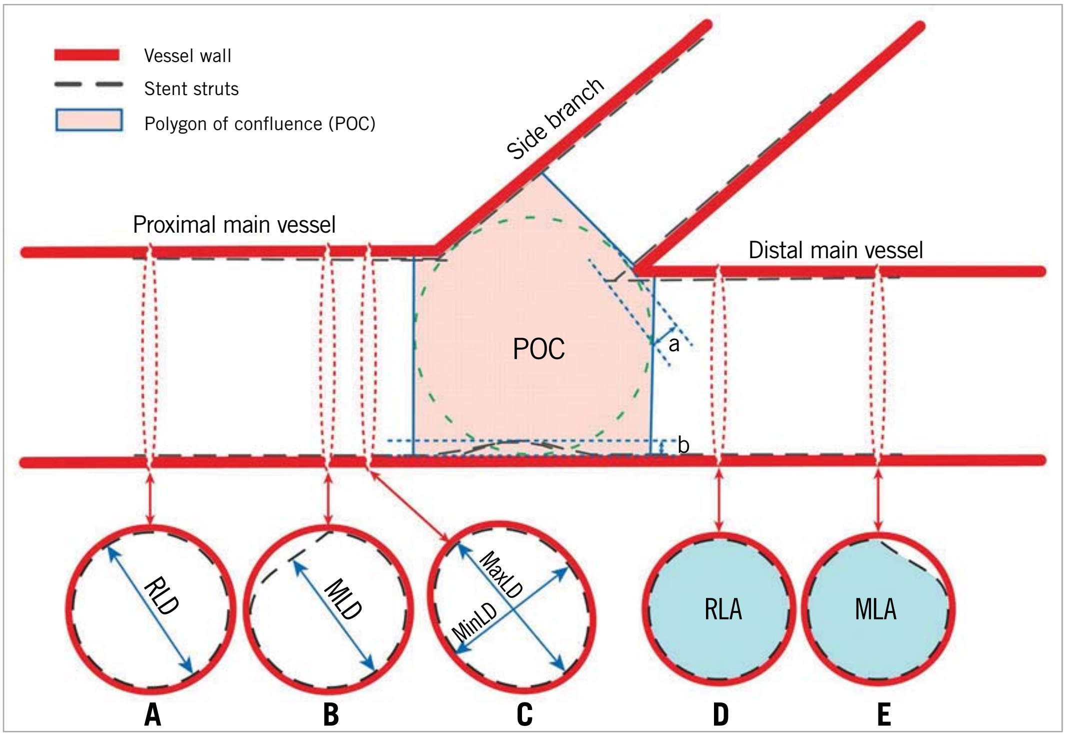

Figure 1. *Schematic diagram for the analysis of bifurcation stenting models. The lengths of a and b represent the metal carina length and maximum distance of malapposed struts for the wall facing the side branch ostium, respectively. Measurement methods of reference lumen diameter (RLD) (A), minimum lumen diameter (MLD) (B), and maximal lumen diameter (MaxLD) and minimal lumen diameter (MinLD) (C) for the proximal main vessel. D) & E) Measurement methods of reference lumen area (RLA) and minimum lumen area (MLA) for the distal main vessel.*

and (1 – MLA/RLA) × 100%, respectively, and the percentage of the SBO area stenosis was equal to (SBO total area - SBO free of strut coverage area)/SBO total area ×100%[12].

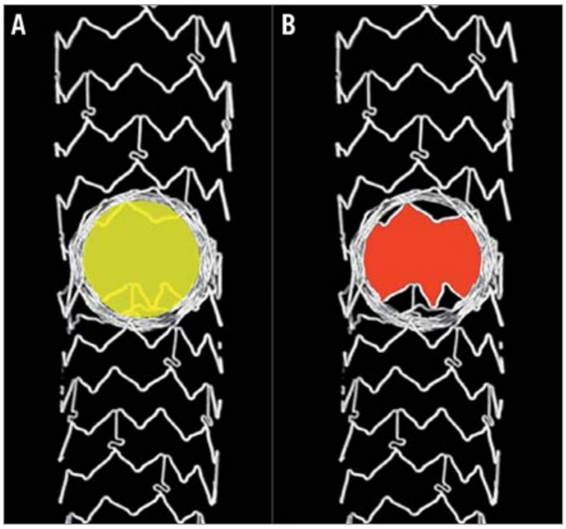

Figure 2. *Measurement methods of side branch ostium area stenosis. The area stenosis was calculated by (area of yellow A – area of red B)/area of yellow A × 100%.*

Moreover, the maximal lumen diameter (MaxLD) and minimal lumen diameter (MinLD) of the cross-section in the asymmetric stent lumen **(Figure 1)** were measured to calculate the symmetry index: symmetry index=MaxLD/MinLD[11].

STATISTICAL ANALYSIS

All statistical tests were performed using SPSS software, Version 22.0 (IBM Corp., Armonk, NY, USA). Continuous variables are presented as mean±standard deviation. Comparisons between the two groups were performed by t-test. If there was a significant difference for the t-test, we performed a factorial analysis to observe the effects of the two bifurcated vessel models. A p-value <0.05 was considered significant, and all tests were two-tailed.

Results

In this study, both DK mini-culotte and mini-culotte bifurcation stenting in Models 1 and 2 were repeated four times, and a total of 32 (16 pairs) Firebird metal stents were used. All deployments were successfully completed, and all bifurcation stenting models were scanned by micro-CT.

We observed that the mini-culotte stenting was associated with obvious strut malapposition, deformation and misdistribution, large blank space (gap), and severe circular stent underexpansion compared to the DK mini-culotte stenting. The underexpansion of

the circular stent at the main vessel stents facing the SBO was more severe in Model 2 than in Model 1 **(Figure 3)**.

When we rotated and dissected the images electronically, stent morphological characteristics such as strut malapposition, deformation and misdistribution, gap, and severe circular stent underexpansion in mini-culotte stenting were more clearly observed **(Figure 4)**.

Furthermore, when the main struts opposite the SBO were electronically removed, we observed that the SBO had more residual strut coverage in the mini-culotte stenting model than in the DK mini-culotte stenting model both in Model 1 and in Model 2 **(Figure 5)**.

To verify our preliminary observational results, quantitative comparisons of the two bifurcation stenting techniques were conducted. We did not find any significant differences in RLD, MLD, RLA, MLA, diameter stenosis, area stenosis, and symmetry index between the two groups at the PMV, DMV or side branch (all p>0.05) **(Table 1)**.

In the polygon of confluence of the bifurcated vascular models, the metal carina length (0.25±0.13 mm vs 0.55±0.15 mm, p=0.023), SBO area stenosis (4.65±3.24% vs 12.5±3.93%, p=0.022) and maximum distance of malapposed struts for the wall facing the SBO (0.27±0.08 mm vs 0.49±0.15 mm, p=0.041) in the DK mini-culotte stenting models were lower than those in the mini-culotte stenting models in Model 1, and the metal carina length (0.21±0.47 mm vs 0.47±0.12 mm, p=0.020), SBO area stenosis (5.13±3.37% vs 15.00±6.18%, p=0.031) and maximum distance of malapposed struts (0.32±0.13 mm vs 0.68±0.10 mm, p=0.004) in the DK mini-culotte stenting models were also lower than those in the mini-culotte stenting models in Model 2 **(Table 1)**.

To analyse the effects of different bifurcation models on stenting techniques, we performed factorial analysis for metal carina length, SBO area stenosis, and maximum distance of malapposed struts (which had significant differences between the two stenting techniques). As for metal carina length and SBO area stenosis, the difference between the two stenting techniques was statistically significant (F=18.873, p=0.001, and F=16.541, p=0.002, respectively), while there was no significant difference between the two kinds of bifurcation model (F=0.810, p=0.386 and F=0.484, p=0.500, respectively). With respect to maximum distance of malapposed struts, the DK mini-culotte stenting technique had better stent apposition than mini-culotte stenting (F=24.507, p=0.000), and the maximum distance of malapposed struts tended to be shorter in Model 1 than in Model 2 (F=4.226, p=0.062). There were no significant interactions between bifurcation stenting models and stenting techniques in the above indicators (all p>0.05).

Discussion

In the present study, we performed bench testing of DK mini-culotte stenting and mini-culotte stenting in a silicone phantom model, and micro-CT was used to evaluate the effects of DK

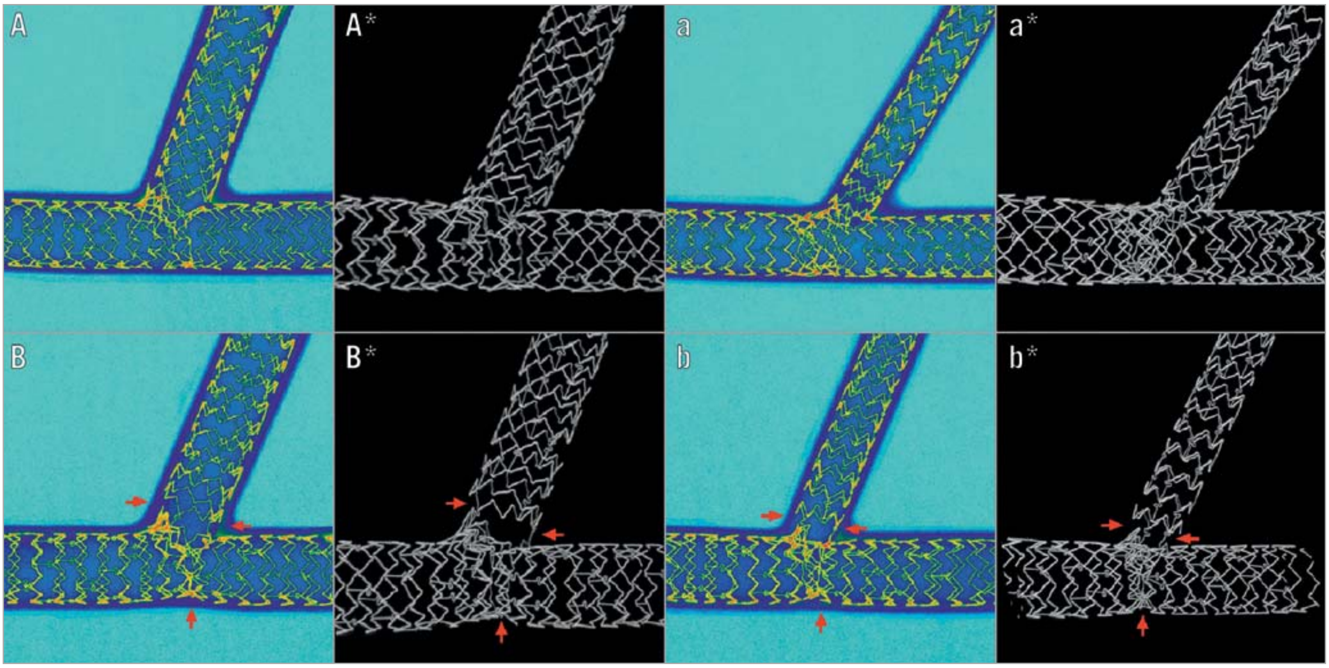

Figure 3. *Micro-computed tomographic images for the double kissing (DK) mini-culotte and mini-culotte stenting phantoms. A) & A*) DK mini-culotte in Model 1. B) & B*) Mini-culotte in Model 1. a) & a*) DK mini-culotte in Model 2. b) & b*) Mini-culotte in Model 2. A, B, a, and b are two-dimensional images by digital processing. Dark blue represents the walls of bifurcated models, light blue represents the phantom lumens, and gold represents the metal struts. A*, B*, a*, and b* are three-dimensional reconstruction images of the stents. Compared with DK mini-culotte stenting, mini-culotte stenting had circular stent underexpansion (vertical arrow) and strut malapposition, deformation, and misdistribution at the side branch ostium (horizontal arrows).*

EuroIntervention 2019;15:465-472

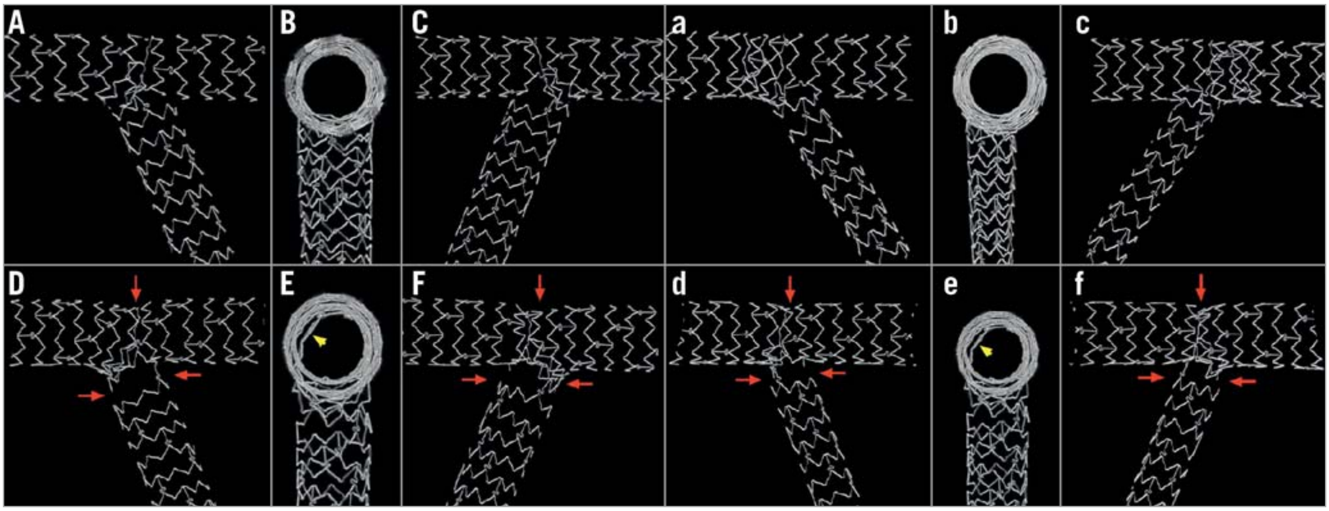

Figure 4. *Cutaway views of the bifurcation stenting phantoms. A), B) & C) Double kissing (DK) mini-culotte in Model 1. D), E) & F) Mini-culotte in Model 1. a), b) & c) DK mini-culotte in Model 2. d), e) & f) Mini-culotte in Model 2. We electronically cut the 3D stenting images along the middle planes of B, E, b, and e. Their left lateral parts produced A, D, a, and d, while C, F, c, and f were their right lateral parts. From the PMV to DMV, underexpanded struts were observed in the mini-culotte stenting models (E and e, arrow). Compared with DK mini-culotte stenting, mini-culotte stenting had circular stent underexpansion (vertical downward arrow), and strut malapposition, deformation and misdistribution at the side branch ostium (horizontal arrows).*

balloon inflation in mini-culotte stenting for the morphologic characteristics of implanted stents. We found that the application of the DK balloon dilation technique in mini-culotte stenting could shorten the metal carina length in the polygon of confluence, improve the circular stent underexpansion at the main vessel stents facing the SBO, enlarge the area of SBO which was free of strut coverage, promote the uniform distribution of struts, and diminish the gap between struts. This evidence showed that the DK mini-culotte stenting technique may be better than mini-culotte stenting.

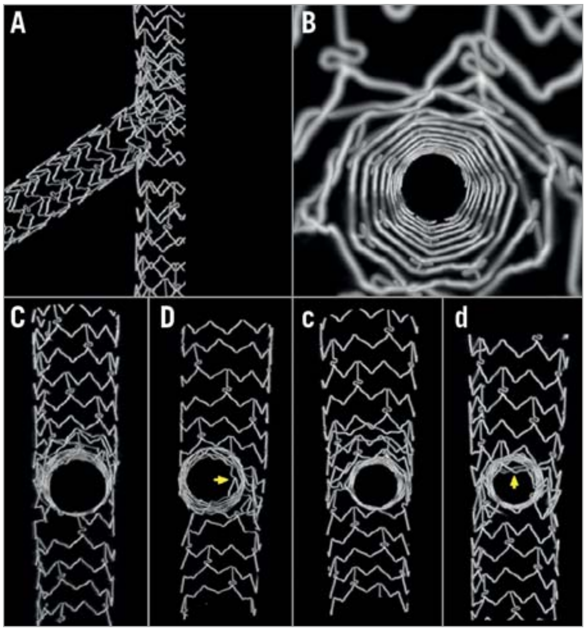

Figure 5. *Morphological features of the side branch ostium (SBO). A) Struts at the opposite side of the SBO were removed digitally. B) Enlarged image from the SBO to side branch. C) DK mini-culotte in Model 1. D) Mini-culotte in Model 1. c) DK mini-culotte in Model 2. d) Mini-culotte in Model 2. When compared with DK mini-culotte stenting models (C and c), mini-culotte stenting (D and d) might have more residual struts across the SBO (arrows).*

The mini technique was an important improvement to the classic double stenting technique. Following the classic crush stenting proposed by Colombo, Galassi et al proposed the mini-crush two-stent technique. They found that mini-crush stenting was simple and associated with lower incidence rates of major adverse cardiovascular events and restenosis[13,14]. In 2011, we first reported the modified mini-culotte stenting technique[7], the main features of which were as follows: stenting the side branch first, emphasising that the protruding part of the side branch stent in the main vessel should be as short as possible (1-3 mm) (mini technique), and embedding a protection balloon in the main branch to reduce the risk of intraoperative vascular occlusion if necessary. The pilot study found that the modified culotte stenting technique had low rates of major adverse cardiovascular events and intravascular restenosis at one-year follow-up.

Although a previous study showed that SBO area stenosis was lower in mini-crush stenting than in crush stenting, the SBO area stenosis in mini-crush stenting was still as high as 36%[12]. The overlapping part of the side branch stent and the main vessel stent in the main vessel was reduced by the mini technique, but the poor morphologic characteristics of the two-stent technique in the polygon of confluence could not be corrected completely. Thus, further improvement of the double stenting technique was necessary.

Table 1. Comparisons of stent morphologic characteristics in DK mini-culotte and mini-culotte stenting phantoms.

Variable	Model 1		*p*-value	Model 2		*p*-value
	DK	Non-DK		DK	Non-DK	
**Proximal main vessel**						
RLD, mm	4.16±0.05	4.14±0.06	0.629	4.17±0.06	4.13±0.04	0.280
MLD, mm	4.03±0.04	3.95±0.06	0.061	4.02±0.05	3.91±0.12	0.127
Diameter stenosis, %	3.16±0.88	4.67±1.58	0.147	3.43±1.37	5.32±2.13	0.187
RLA, mm^2	13.36±0.34	13.24±0.36	0.642	13.43±0.38	13.16±0.27	0.283
MLA, mm^2	12.78±0.12	12.45±0.33	0.107	12.77±0.36	12.31±0.46	0.166
Area stenosis, %	4.32±1.63	5.95±1.07	0.146	4.91±1.57	6.45±1.65	0.225
Symmetry index	1.03±0.02	1.04±0.02	0.261	1.04±0.02	1.05±0.03	0.360
**Distal main vessel**						
RLD, mm	4.04±0.04	4.02±0.04	0.586	4.03±0.04	4.03±0.03	0.922
MLD, mm	3.94±0.06	3.90±0.10	0.532	3.94±0.06	3.89±0.09	0.355
Diameter stenosis, %	2.56±1.04	3.12±1.34	0.533	2.19±1.25	3.38±1.58	0.284
RLA, mm^2	12.58±0.27	12.52±0.27	0.763	12.56±0.23	12.55±0.21	0.938
MLA, mm^2	12.04±0.31	11.88±0.45	0.573	12.07±0.31	11.81±0.44	0.378
Area stenosis, %	4.29±1.44	5.14±1.75	0.481	3.91±1.94	5.86±2.32	0.246
Symmetry index	1.03±0.01	1.04±0.02	0.287	1.03±0.02	1.04±0.01	0.510
**Side branch**						
RLD, mm	3.54±0.06	3.51±0.04	0.473	2.76±0.04	2.75±0.03	0.746
MLD, mm	3.39±0.09	3.32±0.11	0.332	2.64±0.06	2.57±0.03	0.095
Diameter stenosis, %	4.18±1.35	5.68±2.43	0.319	4.39±1.89	6.71±1.93	0.135
RLA, mm^2	9.62±0.26	9.59±0.23	0.848	5.89±0.15	5.86±0.11	0.125
MLA, mm^2	8.97±0.42	8.68±0.52	0.427	5.51±0.20	5.26±0.10	0.067
Area stenosis, %	6.84±2.16	9.48±3.78	0.270	6.59±2.08	10.17±3.45	0.125
Symmetry index	1.03±0.02	1.05±0.13	0.114	1.04±0.02	1.05±0.02	0.325
**Polygon of confluence**						
Metal carina length, mm	0.25±0.13	0.55±0.15	0.023	0.21±0.47	0.47±0.12	0.020
SBO area stenosis, %	4.65±3.24	12.5±3.93	0.022	5.13±3.37	15.00±6.18	0.031
Max distance of malapposed struts, mm	0.27±0.08	0.49±0.15	0.041	0.32±0.13	0.68±0.10	0.004

Values are mean±SD. DK: double kissing; MLA: minimum lumen area; MLD: minimum lumen diameter; RLA: reference lumen area; RLD: reference lumen diameter; SBO: side branch ostium

The kissing balloon dilation technique was another important improvement in the interventional treatment of bifurcation lesions, especially for two-stent techniques. Different studies have shown that final kissing balloon dilatation could reduce strut deformation, correct stent malapposition, optimise the SBO, and improve bifurcated vascular haemodynamic characteristics[15]. However, the final kissing balloon dilatation was not always successfully completed: its success rate was about 95% in the simple strategy and only 85% in the complex strategy and, even if final kissing balloon dilatation was performed, stent malapposition still existed[15,16].

In DK-crush stenting, an increased kissing balloon dilatation was performed after the side branch stent implantation to squeeze the protruding part of the side branch stent fully into the main vessel and effectively clear the struts that covered across the SBO. This procedure reduced the difficulty of rewiring the side branch, increased the success rate and quality of the final kissing balloon dilatation, and improved the haemodynamic characteristics of the vessel lumen[17,18]. A series of clinical studies on DK-crush stenting has shown that DK-crush stenting has better short-term and long-term efficacy, which is superior to the classic crush, culotte and provisional stenting methods in the treatment of true bifurcation lesions[6,19,20].

Based on the above studies, we proposed the DK mini-culotte stenting technique (kissing balloon dilatation was increased before the main branch stent implantation), and the preliminary clinical trial for true bifurcation lesions has been conducted. The results of this trial showed that DK mini-culotte stenting was associated with a lower incidence rate of major adverse cardiovascular events and restenosis than provisional T stenting at one-year follow-up[8].

After stenting, abnormal morphological characteristics such as carina shifting and remodelling, the incomplete stent coverage of the lesions, incomplete stent expansion, stent malapposition and lumen deformation were important reasons for vascular in-stent restenosis and major cardiovascular events[17,21,22].

In this bench study, we found that mini-culotte bifurcation stenting retained malapposition and maldistribution. 1) When the main branch stent expanded **(Supplementary Figure 2G)**, the cell of the side branch stent formed an annular limitation, so that the main branch stent was underexpanded and part of its struts protruded into the main branch lumen, which might lead to thrombus formation within the main vessel and in-stent restenosis. This was the main reason for the limited application of culotte stenting. 2) At the time of inflating the main branch stent **(Supplementary Figure 2G)**, the struts of the side branch stent at the bifurcation carina and ilium were pulled and deformed, causing malapposition, maldistribution and gap. This feature might be an important cause of restenosis and thrombus in the side branch vessel. From the results of the micro-CT analysis, malapposition and maldistribution could not be fully corrected by the subsequent balloon dilation (including the final kissing balloon dilatation). Thus, it was clear that avoiding these problems might be the key to further improvement of culotte stenting. In the current study, we found that in the DK mini-culotte stenting models the side branch stents and their cells were more fully expanded, the side branch stents had better apposition, and the above-mentioned underexpansion, malapposition and maldistribution in mini-culotte stenting were significantly improved. Although this bench testing could not imitate real-world clinical circumstances, DK mini-culotte stenting might have the capacity to improve the technical shortcomings of classic culotte stenting according to the above results.

Of note, the anatomical features of the blood vessel itself such as vessel diameter and coronary bifurcation angle might affect the outcomes of double stenting[23]. The present study shows that the extent of malapposition of the struts in the main vessel opposite the SBO might be more serious when the diameter difference between the main and side branches is larger. This occurs because of the limited expansion of the main branch stent which was constrained by the rewired side branch stent cell and its proximal stent rings (the small diameter stent might have a small-sized stent cell and rings)[8,24]. Thus, the application of culotte stenting requires caution when there is a bifurcation lesion with large mismatch between the main vessel and side branch diameters. The coronary bifurcation angle should also be considered in these two stenting procedures even though we only used a designated angle in this study. When the bifurcation angle is near 90º, the side branch struts protruding into the main branch should not be long, and T stenting could be done well in this situation (without a big gap and superfluous struts); however, if the bifurcation angle is smaller, the side branch stent struts protruding into the main branch should be longer in order to cover the SBO completely.

Limitations

There were several limitations in our study. First, our bifurcation models differed from true vessels in terms of shape, texture, and expansibility, and we did not have the ability to simulate narrow plaques in these models. Furthermore, patients usually have complicated lesions, including severe calcification with circumferential coverage; thus, it was very difficult to reflect fully the real situation of bifurcation lesions in the human body, and the results of the present study are still far from the clinical situation. Lastly, the bifurcation angle and vessel diameter in a true coronary bifurcation are changeable and difficult to imitate. Thus, our results have certain limitations, and the results of this study need to be carefully interpreted.

Conclusions

DK mini-culotte stenting with four technological features (implanting the side branch stent first, embedding a protection balloon if necessary, the mini technique and DK balloon dilatation) was associated with better morphologic characteristics in stent phantoms compared with mini-culotte stenting. It might be associated with better clinical outcomes for patients with true coronary bifurcation lesions.

Impact on daily practice

Compared with mini-culotte stenting, DK mini-culotte stenting was associated with shorter metal carina length, shorter maximum distance of malapposed struts, and smaller SBO area stenosis; therefore, DK mini-culotte stenting may obtain better morphologic characteristics at vascular bifurcation and also lead to good clinical outcome in clinical practice.

Funding

This study was supported by the National Natural Science Foundation of China (No. 81370311 and No. 81670332), Scientific and Technological Project of Henan Province (No. 182102310476), and Youth Foundation of The First Affiliated Hospital of Zhengzhou University (No. YNQN2017109).

Conflict of interest statement

The authors have no conflicts of interest to declare.

References

1. Walsh SJ, Hanratty CG, Watkins S, Oldroyd KG, Mulvihill NT, Hensey M, Chase A, Smith D, Cruden N, Spratt JC, Mylotte D, Johnson T, Hill J, Hussein HM, Bogaerts K, Morice MC, Foley DP. Culotte stenting for coronary bifurcation lesions with 2nd and 3rd generation everolimus-eluting stents: the CELTIC Bifurcation Study. *EuroIntervention.* 2018;14:318-24.

2. Lassen JF, Holm NR, Banning A, Burzotta F, Lefèvre T, Chieffo A, Hildick-Smith D, Louvard Y, Stankovic G. Percutaneous coronary intervention for coronary bifurcation disease: 11th consensus document from the European Bifurcation Club. *EuroIntervention.* 2016;12:38-46.

3. Neumann FJ, Sousa-Uva M, Ahlsson A, Alfonso F, Banning AP, Benedetto U, Byrne RA, Collet JP, Falk V, Head SJ, Jüni P, Kastrati A, Koller A, Kristensen SD, Niebauer J, Richter DJ, Seferovic PM, Sibbing D, Stefanini GG, Windecker S, Yadav R, Zembala MO; ESC Scientific Document Group. 2018 ESC/EACTS Guidelines on myocardial revascularization. *Eur Heart J.* 2019;40:87-165.

4. Ferenc M, Gick M, Comberg T, Rothe J, Valina C, Toma A, Loffelhardt N, Hochholzer W, Riede F, Kienzle RP, Achtari A, Neumann FJ. Culotte stenting

vs TAP stenting for treatment of de-novo coronary bifurcation lesions with the need for side-branch stenting: the Bifurcations Bad Krozingen (BBK) II angiographic trial. *Eur Heart J.* 2016;37:3399-405.

5. Kervinen K, Niemela M, Romppanen H, Erglis A, Kumsars I, Maeng M, Holm NR, Lassen JF, Gunnes P, Stavnes S, Jensen JS, Galloe A, Narbute I, Sondore D, Christiansen EH, Ravkilde J, Steigen TK, Mannsverk J, Thayssen P, Hansen KN, Helqvist S, Vikman S, Wiseth R, Aaroe J, Jokelainen J, Thuesen L; Nordic PCI Study Group. Clinical outcome after crush versus culotte stenting of coronary artery bifurcation lesions: the Nordic Stent Technique Study 36-month follow-up results. *JACC Cardiovasc Interv.* 2013;6:1160-5.

6. Chen SL, Xu B, Han YL, Sheiban I, Zhang JJ, Ye F, Kwan TW, Paiboon C, Zhou YJ, Lv SZ, Dangas GD, Xu YW, Wen SY, Hong L, Zhang RY, Wang HC, Jiang TM, Wang Y, Sansoto T, Chen F, Yuan ZY, Li WM, Leon MB. Clinical Outcome After DK Crush Versus Culotte Stenting of Distal Left Main Bifurcation Lesions: The 3-Year Follow-Up Results of the DKCRUSH-III Study. *JACC Cardiovasc Interv.* 2015,8:1335-42.

7. Chen LL, Fan L, Chen ZY, Zhen XC, Luo YK, Lin CG, Peng YF. Modified culotte stenting for treatment of complex coronary bifurcation lesions: immediate and 9-month outcomes in a pilot study. *Chin Med J (Engl).* 2011;124: 1943-50.

8. Fan L, Chen L, Luo Y, Zhang L, Zhong W, Lin C, Chen Z, Peng Y, Zhen X, Dong X. DK mini-culotte stenting in the treatment of true coronary bifurcation lesions: a propensity score matching comparison with T-provisional stenting. *Heart Vessels.* 2016;31:308-21.

9. Ramcharitar S, Onuma Y, Aben JP, Consten C, Weijers B, Morel MA, Serruys PW. A novel dedicated quantitative coronary analysis methodology for bifurcation lesions. *EuroIntervention.* 2008;3:553-7.

10. Sakata K, Koo BK, Waseda K, Nakatani D, Yock PG, Whitbourn R, Worthley SG, Ormiston J, Webster M, Wilkins GT, Honda Y, Meredith IT, Fitzgerald PJ. A Y-shaped bifurcation-dedicated stent for the treatment of de novo coronary bifurcation lesions: an IVUS analysis from the BRANCH trial. *EuroIntervention.* 2015;10:e1-8.

11. Finet G, Derimay F, Motreff P, Guerin P, Pilet P, Ohayon J, Darremont O, Rioufol G. Comparative Analysis of Sequential Proximal Optimizing Technique Versus Kissing Balloon Inflation Technique in Provisional Bifurcation Stenting: Fractal Coronary Bifurcation Bench Test. *JACC Cardiovasc Interv.* 2015;8:1308-17.

12. Ormiston JA, Webster MW, Webber B, Stewart JT, Ruygrok PN, Hatrick RI. The "crush" technique for coronary artery bifurcation stenting: insights from micro-computed tomographic imaging of bench deployments. *JACC Cardiovasc Interv.* 2008;1:351-7.

13. Galassi AR, Colombo A, Buchbinder M, Grasso C, Tomasello SD, Ussia GP, Tamburino C. Long-term outcomes of bifurcation lesions after implantation of drug-eluting stents with the "mini-crush technique". *Catheter Cardiovasc Interv.* 2007;69:976-83.

14. Galassi AR, Tomasello SD, Capodanno D, Barrano G, Ussia GP, Tamburino C. Mini-crush versus T-provisional techniques in bifurcation lesions: clinical and angiographic long-term outcome after implantation of drug-eluting stents. *JACC Cardiovasc Interv.* 2009;2:185-94.

15. Sgueglia GA, Chevalier B. Kissing balloon inflation in percutaneous coronary interventions. *JACC Cardiovasc Interv.* 2012;5:803-11.

16. Foin N, Torii R, Mortier P, De Beule M, Viceconte N, Chan PH, Davies JE, Xu XY, Krams R, Di Mario C. Kissing balloon or sequential dilation of the side branch and main vessel for provisional stenting of bifurcations: lessons from micro-computed tomography and computational simulations. *JACC Cardiovasc Interv.* 2012;5:47-56.

17. Chen SL, Mintz G, Kan J, Zhang JJ, Hu ZY, Ye F, Tian NL, Zhang JX, Xu T, Liu ZZ. Serial intravascular ultrasound analysis comparing double kissing and classical crush stenting for coronary bifurcation lesions. *Catheter Cardiovasc Interv.* 2011;78:729-36.

18. Chen SL, Kan J, Zhang JJ, Hu ZY, Xu T. Hemodynamic change in wall shear stress in patients with coronary bifurcation lesions treated by double kissing crush or single-stent technique. *Chin Med J (Engl).* 2012;125:1720-6.

19. Chen SL, Zhang JJ, Han Y, Kan J, Chen L, Qiu C, Jiang T, Tao L, Zeng H, Li L, Xia Y, Gao C, Santoso T, Paiboon C, Wang Y, Kwan TW, Ye F, Tian N, Liu Z, Lin S, Lu C, Wen S, Hong L, Zhang Q, Sheiban I, Xu Y, Wang L, Rab TS, Li Z, Cheng G, Cui L, Leon MB, Stone GW. Double Kissing Crush Versus Provisional Stenting for Left Main Distal Bifurcation Lesions: DKCRUSH-V Randomized Trial. *J Am Coll Cardiol.* 2017;70:2605-17.

20. Chen SL, Santoso T, Zhang JJ, Ye F, Xu YW, Fu Q, Kan J, Zhang FF, Zhou Y, Xie DJ, Kwan TW. Clinical Outcome of Double Kissing Crush Versus Provisional Stenting of Coronary Artery Bifurcation Lesions: The 5-Year Follow-Up Results From a Randomized and Multicenter DKCRUSH-II Study (Randomized Study on Double Kissing Crush Technique Versus Provisional Stenting Technique for Coronary Artery Bifurcation Lesions). *Circ Cardiovasc Interv.* 2017;10:e004497.

21. Gwon HC, Song YB, Pan M. The story of plaque shift and carina shift. *EuroIntervention.* 2015;11 Suppl V:V75-7.

22. Gomez-Lara J, Salvatella N, Gonzalo N, Hernández-Hernández F, Fernandez-Nofrerias E, Sánchez-Recalde A, Bastante T, Marcano A, Romaguera R, Ferreiro JL, Roura G, Teruel L, Ariza-Solé A, Miranda-Guardiola F, Rodríguez García-Abad V, Gomez-Hospital JA, Alfonso F, Cequier A. IVUS-guided treatment strategies for definite late and very late stent thrombosis. *EuroIntervention.* 2016;12:e1355-65.

23. Tiroch K, Mehilli J, Byrne RA, Schulz S, Massberg S, Laugwitz KL, Vorpahl M, Seyfarth M, Kastrati A; ISAR-LEFT MAIN Study Investigators. Impact of coronary anatomy and stenting technique on long-term outcome after drug-eluting stent implantation for unprotected left main coronary artery disease. *JACC Cardiovasc Interv.* 2014;7:29-36.

24. Erglis A, Lassen JF, Di Mario C. Technical aspects of the culotte technique. *EuroIntervention.* 2015;11 Suppl V:V99-101.

Supplementary data

Supplementary Figure 1. The main steps of double kissing (DK) mini-culotte stenting in a silicone phantom.

Supplementary Figure 2. The main steps of mini-culotte stenting in a silicone phantom.

The supplementary data are published online at: https://eurointervention.pcronline.com/ doi/10.4244/EIJ-D-18-00688

Supplementary data

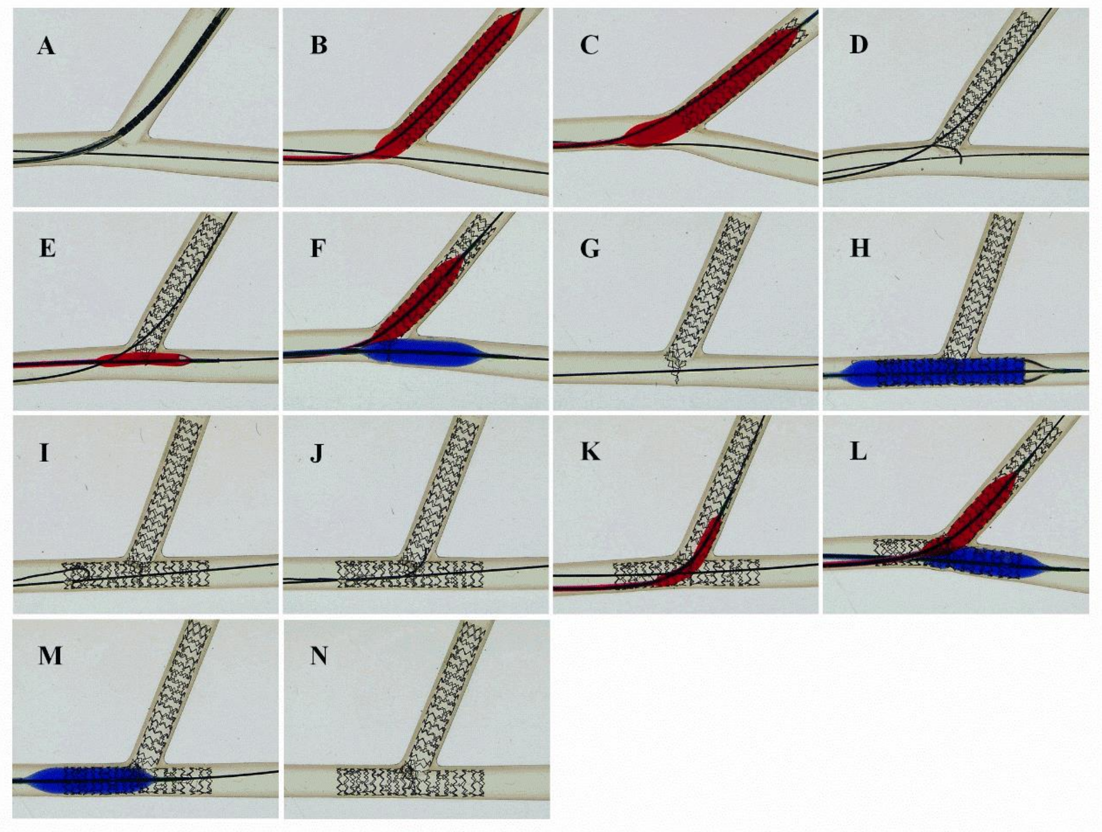

Supplementary Figure 1. The main steps of DK mini-culotte stenting in a silicone phantom.

A) Positioning the side branch (SB) stent with 1-3 mm struts protruding into the main branch (MB) after wiring both branches with two different guidewires.
B) The first stent was deployed at 9 atm (nominal pressure).
C) Balloon dilatation at 12 atm after slightly retracting (2-3 mm) the deflated SB stent balloon.
D) After removing the stent balloon, a third guidewire was used to recross the struts of the first stent (through the distal cell which was adjacent to the carina) into the distal vessel bed of the MB.
E) Pre-expanding the cell of the SB stent with a small-sized balloon after removing the previous jailed MB guidewire.
F) Positioning two short and 1:1 diameter-matched non-compliant (NC) balloons to the SB and MB. After inflating the two balloons at 16 atm, the first kissing balloon inflation was performed in a sequential manner, both at 10 atm (MB post-dilation was performed after SB dilation), to open the cell adequately and prepare the passage of the MB stent.
G) Removing the balloons and SB guidewire (preventing metal-to-metal jail).
H) MB stent diameter was selected according to the distal MB diameter. Positioning the MB stent through the SB stent cell, and deploying this stent at 9 atm.
I) Guiding a U-shaped wire into the MB stent (proximal optimisation technique was recommended to facilitate SB access after the second stent implantation).
J) Rewiring the SB through the distal cell adjacent to the carina.
K) Pre-expansion of the strut cell at the SB ostium with a small-sized balloon.
L) After inflating each branch balloon at 16 atm, final kissing balloon inflation was performed with the two matched NC balloons in a sequential manner, both at 12 atm.
M) Proximal optimisation technique by inflating the MB short NC balloon (this balloon should be sufficiently short to avoid inflation outside the stent) at 20 atm.
N) Final results after removal of the balloons and guidewires.

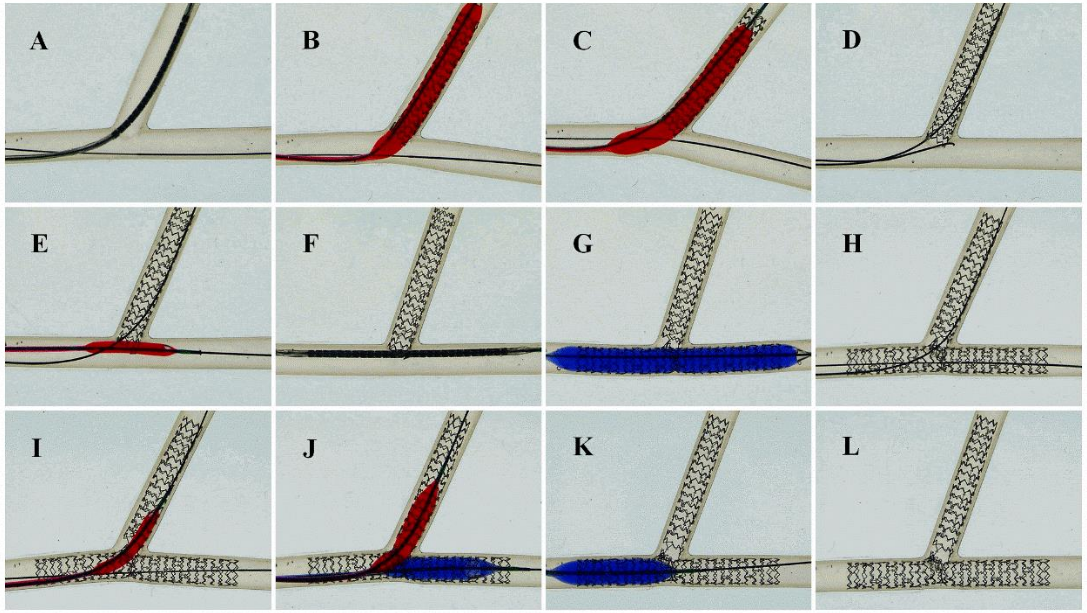

Supplementary Figure 2. The main steps of mini-culotte stenting in a silicone phantom.

A) Positioning the side branch (SB) stent with 1-3 mm struts protruding into the main branch (MB) after wiring both branches with two different guidewires.

B) The first stent was deployed at 9 atm (nominal pressure).

C) Balloon dilatation at 12 atm after slightly retracting (2-3 mm) the deflated SB stent balloon.

D) After removing the stent balloon, a guidewire was used to recross the struts of the first stent (through the distal cell adjacent to the carina) into the distal vessel bed of the MB.

E) Pre-expanding the cell of the SB stent with a small-sized balloon.

F) Positioning the MB stent through the SB stent cell (MB stent diameter was selected according to the distal MB diameter).

G) The MB stent was deployed at 9 atm.
H) Rewiring the SB through the distal cell adjacent to the carina.
I) Pre-expansion of the strut cell at the SB ostium with a small-sized balloon.
J) Final kissing balloon inflation was performed with the two matched NC balloons in a sequential manner, both at 12 atm after inflating each branch balloon at 16 atm.
K) Proximal optimisation technique by inflating the MB short NC balloon at 20 atm.
L) Final results after removal of the balloons and guidewires.
